# Obstructive Sleep Apnea and Pathological Characteristics of Resected Pancreatic Ductal Adenocarcinoma

**DOI:** 10.1371/journal.pone.0164195

**Published:** 2016-10-12

**Authors:** Marco Dal Molin, Aaron Brant, Amanda L. Blackford, James F. Griffin, Koji Shindo, Thomas Barkley, Neda Rezaee, Ralph H. Hruban, Christopher L. Wolfgang, Michael Goggins

**Affiliations:** 1 Department of Pathology, The Sol Goldman Pancreatic Cancer Research Center, The Johns Hopkins University School of Medicine, Baltimore, Maryland, United States of America; 2 Department of Oncology, The Sol Goldman Pancreatic Cancer Research Center, The Johns Hopkins University School of Medicine, Baltimore, Maryland, United States of America; 3 Department of Surgery, The Sol Goldman Pancreatic Cancer Research Center, The Johns Hopkins University School of Medicine, Baltimore, Maryland, United States of America; 4 Department of Medicine, The Sol Goldman Pancreatic Cancer Research Center, The Johns Hopkins University School of Medicine, Baltimore, Maryland, United States of America; Centro Nacional de Investigaciones Oncologicas, SPAIN

## Abstract

**Background:**

Prospective studies have identified obstructive sleep apnea (OSA) as a risk factor for increased overall cancer incidence and mortality. The potential role of OSA in the risk or progression of specific cancers is not well known. We hypothesized that pathological differences in pancreatic cancers from OSA cases compared to non-OSA cases would implicate OSA in pancreatic cancer progression.

**Methods:**

We reviewed the medical records of 1031 patients who underwent surgical resection without neoadjuvant therapy for pancreatic ductal adenocarcinoma (PDAC) at Johns Hopkins Hospital between 2003 and 2014 and compared the TNM classification of their cancer and their overall survival by patient OSA status.

**Results:**

OSA cases were significantly more likely than non-OSA cases to have lymph node-negative tumors (37.7% vs. 21.8%, *p* = 0.004). Differences in the prevalence of nodal involvement of OSA vs. non-OSA cases were not associated with differences in other pathological characteristics such as tumor size, tumor location, resection margin status, vascular or perineural invasion, or other comorbidities more common to OSA cases (BMI, smoking, diabetes). A logistic regression model found that a diagnosis of OSA was an independent predictor of lymph node status (hazard ratio, 0.051, p = 0.038). Patients with OSA had similar overall survival compared to those without OSA (HR, 0.89, (0.65–1.24), p = 0.41).

**Conclusion:**

The observed pathological differences between OSA-associated and non-OSA-associated pancreatic cancers supports the hypothesis that OSA can influence the pathologic features of pancreatic ductal adenocarcinoma.

## Introduction

Pancreatic ductal adenocarcinoma (PDAC) is increasing in incidence and is expected to become the 2^nd^ leading cause of cancer death in the USA by 2020 [1], yet the factors responsible for this rising incidence are not well understood. The rising incidence of metabolic diseases associated with obesity, diabetes and physical inactivity are suspected to be important, as well as increased aging of the population, but other risk factors may be important. One potential cancer risk factor is obstructive sleep apnea (OSA), which has been identified as a risk factor for overall cancer risk and mortality in cohort studies [2–5], although not all studies have found an association [6]. OSA is a common, under-diagnosed disorder [7] characterized by episodes of nocturnal upper airway obstruction, oxygen desaturation, and disrupted sleep (e.g. nocturnal gasping, choking, witnessed apneic events), and is associated with numerous comorbidities, including hypertension, diabetes mellitus, metabolic syndrome and daytime sleepiness. Patients with OSA have an increased risk of cardiovascular and cerebrovascular mortality [3]. In addition to reports evaluating overall cancer incidence and mortality, one recent study evaluating the role of OSA on a patient’s melanoma progression found that the presence and severity of OSA was associated with faster tumor growth and other measures of tumor aggressiveness [8]. Reports of potential mechanisms by which OSA might have oncogenic effects have focused on the signaling pathways such as HIF-1alpha [9] induced by hypoxia, the role of intermittent hypoxia in promoting oxidative stress-induced DNA damage [10], and the promotion of tumor development through the creation of a pro-inflammatory [11], immunosuppressive microenvironment [12, 13]. The microenvironment of a cancer is already known to create immunosuppression and it is thought that hypoxia contributes to this; indeed in preclinical cancer models immunosuppressive characteristics of the tumor microenvironment can be reversed by supplemental oxygen [14]. There is also increasing interest in the role a pro-inflammatory and immunosuppressive tumor microenvironment has in promoting pancreatic cancer [15]. OSA could also promote tumorigenesis through systemic metabolic effects [16]; pancreatic cancer risk is increased in patients with obesity and long-standing diabetes [17], markers of the metabolic syndrome which is very commonly found in patients with OSA [18, 19].

Diagnosing OSA in the clinical setting first requires that clinicians suspect their patient might have OSA and order a sleep study [20]. A diagnostic sleep study is needed to establish an OSA diagnosis, one that demonstrates obstructive respiratory events during sleep, i.e. apnea and/or hypopnea associated an absence of respiratory airflow despite ventilatory effort (>5 events/hour) [20] [21]. Because determining the prevalence of OSA in a population requires evaluating subjects for these clinical features and performing sleep studies on large numbers of individuals, there have been few large prospective population-based studies designed to investigate the future cancer risks associated with having OSA and none of these studies have been large enough to quantify the risk of individual cancers such as pancreatic cancer.

In the absence of such studies, the identification of pathological differences in the pancreatic cancers from patients with OSA compared to those without OSA would support the hypothesis that OSA influences the pathogenesis of pancreatic cancer.

## Materials & Methods

### Patients

We evaluated the clinicopathological characteristics and survival of 1031 patients who underwent surgical resection of their primary pancreatic ductal adenocarcinoma at the Johns Hopkins Hospital (2003–2014). The study period was selected to obtain a total sample size of ~ 1000 cases with an estimated OSA prevalence of between 5–10%, as suggested by epidemiological studies[22, 23] [7]. Cases were enrolled into our study protocol during their hospital admission in the pre-operative period because of their diagnosis of pancreatic cancer. TNM staging of the resected primary pancreatic cancer was performed by surgical pathologists with expertise in pancreatic pathology. Clinical, pathological and demographic data, as well as follow up and survival data were retrieved from a prospectively collected surgical database and from patient clinical records and were ascertained in the same manner for OSA and non-OSA cases. Patients with variant forms of pancreatic ductal adenocarcinoma such as colloid cancers arising from an intraductal papillary mucinous neoplasm or a mucinous cystic neoplasm were excluded as these cancers have biological differences and a different natural history than usual pancreatic ductal adenocarcinoma. To eliminate potential confounding effects of therapy on TNM staging, patients who had received neoadjuvant therapy were excluded. Since many patients who undergo pancreatic resection at our institution receive their post-op chemoradiotherapy elsewhere, follow up information was not available for all cases. Written consent was obtained and saved from all subjects. This study was approved by the Johns Hopkins University Institutional Review Board.

### Identification of cases with obstructive sleep apnea (OSA)

Patients were included in the OSA group if a diagnosis of OSA was documented by a positive polysomnography that was either directly available for review, or reported in the medical record. Whenever information on previous polysomnographic studies was not available, patients were considered positive for OSA if a diagnosis of OSA was reported in the anesthesia records and/or elsewhere in the preoperative medical record. Information about current or past therapy with either continuous positive airway pressure (CPAP) or other devices to treat OSA was considered a surrogate indicator of OSA. “Two patients that self-reported OSA based only on symptoms without having undergone any diagnostic sleep study were excluded from analysis because we could not confirm the diagnosis of OSA in the cases. All patients not known to have a history of OSA were included in the non-OSA group. This included all patients with other symptoms of sleep disturbances such as snoring in the absence of witnessed episodes of apnea since prior studies have found that snoring in the absence of other symptoms associated with sleep apnea is not likely to be due to sleep apnea [20]. Similarly, patients without an OSA diagnosis but with other chronic respiratory diseases such as chronic obstructive pulmonary disease (COPD) were not excluded.

### Pathological characteristics

Pathological data were retrieved from the prospectively maintained Johns Hopkins Hospital surgical pathology database and updated whenever necessary to comply with the 7^th^ edition of the American Joint Committee on Cancer (AJCC) classification.

### Statistical analyses

The most important pathological variables in the pathology reports we wanted to compare between the OSA and non-OSA cases were those that are the best independent predictors of prognosis and that are most likely to reflect differences in underlying pathology, i.e. tumor grade, tumor size and lymph node status. Continuous variables are presented as median and range and compared using the Wilcoxon rank sum tests. Categorical variables are presented as frequencies and compared using the Fisher exact test or the Chi-square test, when appropriate. Multivariable logistic regression models were used to test for interactions between OSA and nodal status on clinicopathological outcomes and to identify factors associated with nodal status. Overall survival was defined as date of surgery to date of death or 04/15/2015. Hazard ratios (HR) and median survival were estimated using the multivariable Cox Proportional Hazards model, adjusting for patient age, adjuvant chemotherapy and radiation therapy, tumor margin status and stratifying by lymph node status. Patients who had experienced mortality within 30 days from surgical resection were excluded from survival analysis, as death in those individuals was most likely secondary to surgical complications. There are no adjustments to the significance values due to multiple comparisons, as all p-values are presented for descriptive purposes only. All statistical analyses were performed using R (R Core Team-2014; R Foundation for Statistical Computing, Vienna, Austria, http://www.R-project.org)

## Results

Sixty-nine of the 1031 patients (6.7%) had received an OSA diagnosis prior to their pancreatic cancer diagnosis. Of the 69 cases diagnosed with OSA, 39 had documentation of sleep studies confirming the diagnosis, 37 of these also had evidence of OSA treatment such as CPAP, another 11 had documentation that their OSA had not been treated either because the patient refused treatment or because the OSA was considered mild, and the remaining 21 cases had a diagnosis of OSA recorded in the medical record without any additional information. The percentage of cases with an OSA diagnosis increased from 3.1% of PDAC cases who underwent pancreatic resection in the years 2003–2007 to 10.8% of cases diagnosed between 2008 and 2014. To account for year of diagnosis as a potential confounder, we also performed a subgroup analysis of cases diagnosed since 2008 (since ~80% of the OSA cases in this series were diagnosed since 2008). Patient characteristics for the total study population are presented in Table 1. Patients with OSA were more often male (76.8% vs. 50.8%, *p*<0.001), diabetic (31.9% vs. 21.1%; *p* = 0.049), and had a higher BMI than patients without OSA (median 27.2 kg/m^2^ vs. 24.9 kg/m^2^; *p*<0.001). OSA cases trended towards a higher prevalence of cardiovascular and cerebrovascular events prior to their pancreatic cancer diagnosis (p = 0.085). The prevalence of the presenting symptoms of preoperative jaundice and weight loss was similar between OSA and non-OSA cases.

**Table 1:** Characteristics of all subjects, by whether or not patients had obstructive sleep apnea (OSA).

	non-OSA	OSA	*p*-value*
	N = 962	N = 69	
Race—no. (%)			
African American	61 (6.3)	5 (7.2)	0.655
Other	89 (9.3)	4 (5.8)	
Caucasian	812 (84.4)	60 (87)	
Sex—no. (%)			
Female	473 (49.2)	16 (23.2)	< 0.001
Male	489 (50.8)	53 (76.8)	
Age—median (range)	67 (27, 92)	67 (53, 84)	0.768
Weight (kg)—median (range)	72.2 (36.3, 147)	84.3 (55.7, 156)	< 0.001
Height (m)—median (range)	1.7 (1.4, 2)	1.8 (1.4, 1.9)	0.002
BMI—median (range)	24.9 (15.1, 46.2)	27.2 (18.7, 52.3)	< 0.001
Smoking Status—no. (%)			
Non-smoker	357 (45.6)	26 (38.8)	0.511
Current	73 (9.3)	6 (9)	
Former	353 (45.1)	35 (52.2)	
Unknown	179	2	
Diabetes—no. (%)	203 (21.1)	22 (31.9)	0.049
Cardiovascular/cerebrovascular events—no. (%)	81 (8.4)	10 (14.5)	0.085
Pre-Op Jaundice—no. (%)	500 (52)	31 (45.6)	0.318
Pre-Op Weight Loss—no. (%)	366 (38)	21 (31.3)	0.299
Tumor location—no. (%)			
Body-Tail	150 (15.6)	18 (26.1)	0.02
Head	769 (79.9)	51 (73.9)	
Whole Gland	43 (4.5)	0 (0)	
Tumor size—median (range)	3 (0.7, 9.5)	3.1 (0.8, 10)	0.699
Path Grade—no. (%)			
1	36 (3.8)	3 (4.4)	0.877
2	515 (53.8)	37 (54.4)	
3	407 (42.5)	28 (41.2)	
Unknown	4	1	
Vascular Invasion—no. (%)	506 (62.6)	39 (61.9)	0.893
Perineural Invasion—no. (%)	856 (91)	63 (91.3)	>0.99
Positive Nodes—median (range)	2 (0, 20)	1 (0, 16)	0.004
Total Nodes—median (range)	20 (0, 84)	19 (3, 39)	0.94
Nodal Ratio—median (range)	0.1 (0, 2)	0.1 (0, 0.8)	0.004
AJCC T Stage—no. (%)			
T1	66 (6.9)	9 (13)	0.029
T2	212 (22)	22 (31.9)	
T3	655 (68.1)	36 (52.2)	
T4	29 (3)	2 (2.9)	
AJCC N Stage—no. (%)			
N0	210 (21.8)	26 (37.7)	0.004
N1	752 (78.2)	43 (62.3)	
AJCC N Stage—no. (%) Tumor location head only			
N0	143 (18.6)	16 (31.4)	0.025
N1	626 (81.4)	35 (68.6)	
Stage—no. (%)			
IA	38 (4)	5 (7.2)	0.004
IB	70 (7.3)	14 (20.3)	
IIA	99 (10.3)	7 (10.1)	
IIB	726 (75.5)	41 (59.4)	
III	29 (3)	2 (2.9)	
Margin Status—no. (%)			
R0	658 (69.3)	50 (72.5)	0.573
R1	269 (28.3)	19 (27.5)	
R2	23 (2.4)	0 (0)	
Unknown	12	0	
Adjuvant chemotherapy—no. (%)	521 (54.2)	41 (59.4)	0.453
Adjuvant radiation therapy—no. (%)	336 (34.9)	24 (34.8)	>0.99

* P-values for Fisher's exact test for categorical variables and Wilcoxon rank sum tests for continuous variables.

The median primary tumor size of OSA cases (3.1 cm) was similar to non-OSA cases (3.0 cm) (Table 1), yet cases with OSA were more likely than non-OSA cases to be free of lymph node metastases (37.7% vs. 21.8%, *p* = 0.004). This association was still significant if we restricted the OSA cases to those with documentation of a positive sleep study (46% node-negative, p = 0.001). (This association was not explained by lymph node sampling as the number of lymph nodes analyzed was similar in OSA vs. non-OSA cases overall (Table 1) and in the OSA vs. non-OSA cases without lymph node involvement (data not shown). In addition, the lymph node ratio (the ratio of nodes positive for metastases to total sampled nodes was significantly lower in the OSA compared to the non-OSA group (Table 1). This lower prevalence of nodal involvement among OSA cases was also true for the subgroup of OSA subjects who had been receiving OSA treatment (48.1% of whom had node-negative disease compared to the 21.8% with node-negative disease in the non-OSA group, p<0.0001). There was no significant difference in tumor size or other pathological variables between OSA subjects who had received treatment vs. those who had not, although this comparison was limited by small sample size. The primary tumors of OSA cases also had lower average T-stage and thus lower average TNM stage than non-OSA cases (*p* = 0.004). OSA cases were more likely to have lower T-stage tumors despite having similar average tumor size to non-OSA cases because there were fewer T3 tumors in OSA cases and an increase in T1 and T2 cases (the T3 stage is defined by the presence of invasion into adjacent organs). The proportion of T3 cases was 68.1% in non-OSA cases vs. 52.2% in OSA cases, p = 0.006). This association with T-stage and proportion of T3 cases also remained significant if OSA cases were restricted to those with documentation of a positive sleep study (both, p = 0.028). There was no difference between OSA and non-OSA cases with respect to tumor grade, perineural or vascular invasion, or margin status.

To investigate clinicopathological factors associated with having lymph node metastases, we compared patient and tumor characteristics by their lymph node involvement (Table 2). As expected, patients with “node-negative” cancers were more likely than “node-positive” cases to have smaller tumors (2.6 cm vs. 3.0 cm, respectively; *p*<0.001), with lower T-stage (*p*<0.001). Consistent with having smaller cancers overall, patients with node-negative PDAC were less likely to have jaundice (34.3% vs. 56.7%, *p*<0.001), vascular (25.5% vs. 74.2%; *p*<0.001), perineural invasion (85.2% vs. 92.7%; *p*<0.001), or positive resection margins (18.2% vs. 34.2%, *p*<0.001). No other differences in demographic and patient characteristics were identified.

**Table 2:** Characteristics of all subjects, by nodal status.

	Node Negative Patients	Node Positive Patients	*p*-value*
	N = 236	N = 795	
Race—no. (%)			
African American	21 (8.9)	45 (5.7)	0.11
Other	25 (10.6)	68 (8.6)	
Caucasian	190 (80.5)	682 (85.8)	
Sex—no. (%)			
Female	118 (50)	371 (46.7)	0.409
Male	118 (50)	424 (53.3)	
Age—median (range)	68 (35, 91)	67 (27, 92)	0.081
Weight (kg)—median (range)	73.7 (42.3, 144.4)	72.7 (36.3, 156)	0.432
Height (m)—median (range)	1.7 (1.4, 1.9)	1.7 (1.5, 2)	0.734
BMI—median (range)	25.5 (17.3, 45.3)	24.8 (15.1, 52.3)	0.075
Smoking Status—no. (%)			
Non-smoker	88 (45.6)	295 (44.9)	0.983
Current	18 (9.3)	61 (9.3)	
Former	87 (45.1)	301 (45.8)	
Unknown	43	138	
Diabetes—no. (%)	50 (21.2)	175 (22)	0.857
Cardiovascular/cerebrovascular events—no. (%)	25 (10.6)	66 (8.3)	0.276
Pre-Op Jaundice—no. (%)	81 (34.3)	450 (56.7)	< 0.001
Pre-Op Weight Loss—no. (%)	80 (34)	307 (38.7)	0.227
Tumor location—no. (%)			
Body-Tail	66 (28)	102 (12.8)	< 0.001
Head	159 (67.4)	661 (83.1)	
Whole Gland	11 (4.7)	32 (4)	
Tumor size—median (range)	2.6 (0.7, 10)	3 (0.7, 9.5)	< 0.001
Path Grade—no. (%)			
1	17 (7.2)	22 (2.8)	0.002
2	132 (56.2)	420 (53.1)	
3	86 (36.6)	349 (44.1)	
Unknown	1	4	
Vascular Invasion—no. (%)	53 (25.5)	492 (74.2)	< 0.001
Perineural Invasion—no. (%)	196 (85.2)	723 (92.7)	< 0.001
Total Nodes—median (range)	18 (0, 51)	20 (1, 84)	< 0.001
AJCC T Stage—no. (%)			
T1	43 (18.2)	32 (4)	< 0.001
T2	84 (35.6)	150 (18.9)	
T3	106 (44.9)	585 (73.6)	
T4	3 (1.3)	28 (3.5)	
Margin Status—no. (%)			
R0	189 (81.8)	519 (65.9)	< 0.001
R1	40 (17.3)	248 (31.5)	
R2	2 (0.9)	21 (2.7)	
Unknown	5	11	
Adjuvant chemotherapy—no. (%)	121 (51.3)	441 (55.5)	0.288
Adjuvant radiation therapy—no. (%)	67 (28.4)	293 (36.9)	0.02

*p-values for Chi-square tests for categorical variables and Wilcoxon rank sum tests for continuous variables.

Node-negative pancreatic cancers were also more likely to be located in the body or tail of the gland (28.0% vs. 12.8%, *p*<0.001), as has been reported previously [24]. Notably, the reduced odds of having lymph node metastases among OSA cases remained significant when the analysis was restricted to subjects with pancreatic head and/or uncinate cancers (*p* = 0.025) and this association remained significant when we restricted OSA cases to those with documented sleep studies (p = 0.024)(Table 1).

A logistic regression model of all PDAC cases showed no significant interaction between OSA and nodal status, suggesting that clinicopathological differences identified between OSA and non-OSA cases are not driven by nodal status (S1 Table). Comorbidities more common to patients with OSA (obesity, diabetes, and smoking) were not associated with the pathological patterns such as lymph node status observed in OSA cases (data not shown). To address the possibility that period of diagnosis could have influenced our results, we performed a subgroup analysis of PDAC cases diagnosed from 2008–14. This analysis also found OSA cases were significantly more likely to have node-negative and lower T-stage cancers (S2 Table).

We performed a logistic regression model to identify factors independently associated with nodal status (Table 3). Tumor size, tumor location, vascular invasion and having OSA (hazard ratio 0.51, 95% CI 0.27 to 0.96, p = 0.038) were all independently associated with lymph node status.

**Table 3:** Odds ratios from a logistic regression model examining the association between OSA status and lymph node status (positive versus negative), adjusting for other clinical factors.

	Odds Ratio	95% CI	P
OSA v. No OSA	0.51	(0.27, 0.96)	0.038
Vascular Invasion	7.11	(4.92, 10.29)	< 0.001
Tumor Size	1.5	(1.27, 1.76)	< 0.001
Head v. Body/Tail	3.12	(1.97, 4.95)	< 0.001
Whole Gland v. Body/Tail	2.01	(0.78, 5.2)	0.15

**Fig 1:**
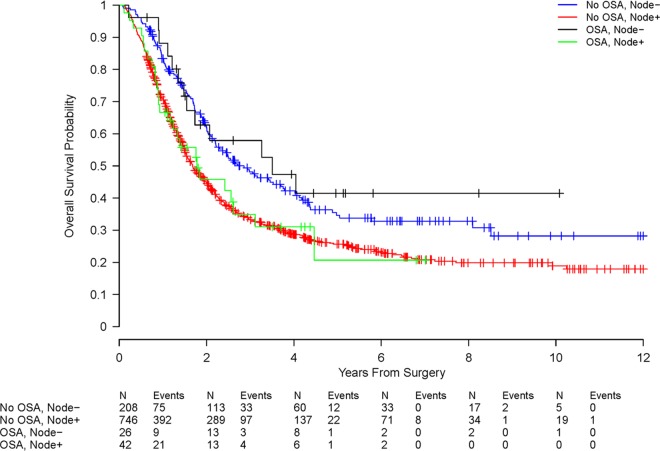
Kaplan-Meir curves illustrating overall patient survival among patients with pancreatic ductal adenocarcinoma with and without a diagnosis of obstructive sleep apnea stratified by nodal status.

We also examined survival in our study population. Node positive patients had worse OS than node negative patients (HR 1.52, 95% CI 1.25 to 1.84, p < 0.0001), and this association was similar within OSA patients (HR 1.63, 95% CI 0.83 to 3.17) and non-OSA patients (HR 1.51, 95% CI 1.23 to 1.84) (Table 4). Survival did not differ between OSA patients and non-OSA patients overall (HR 0.89, 95% CI 0.65 to 1.24, p = 0.50), nor within node-negative patients (HR 0.91 95% CI 0.51, 1.62, p = 0.75) or node-positive patients (HR 0.98, 95% CI 0.66 to 1.46, p = 0.94). Fig 1 presents the Kaplan-Meier curves for overall survival among the OSA vs. non-OSA patients stratified by nodal status.

**Table 4:** Overall survival (OS) of patients, by OSA status, nodal status and a combination of the two.

	N	Median OS	1y OS	2y OS	5y OS	HR	95% CI	*p-value*
non-OSA	954	1.94 [1.72, 2.09]	74 [71, 76]	49 [45, 52]	28 [25, 31]	1	-	
OSA	68	2.42 [1.55, 4.46]	75 [65, 86]	52 [41, 67]	31 [20, 48]	0.87	(0.63,1.21)	0.41
N-	234	2.94 [2.22, 4.02]	84 [79, 89]	62 [56, 69]	35 [29, 43]	1	-	
N+	788	1.69 [1.54, 1.94]	71 [68, 74]	45 [42, 49]	26 [22, 29]	1.61	(1.33,1.94)	< 0.0001
non-OSA, N-	208	2.86 [2.21, 4.02]	83 [78, 89]	62 [55, 69]	35 [28, 43]	1	-	
non-OSA, N+	746	1.68 [1.54, 1.94]	71 [68, 74]	45 [41, 49]	26 [22, 29]	1.59	(1.3,1.94)	< 0.0001
OSA, N-	26	3.5 [1.73, 12+]	88 [76, 100]	63 [46, 86]	41 [25, 69]	0.89	(0.5,1.58)	0.7
OSA, N+	42	1.78 [1.24, 12+]	67 [54, 83]	46 [32, 65]	21 [8, 52]	1.56	(1.02,2.38)	0.04
non-OSA, N+	746	1.68 [1.54, 1.94]	71 [68, 74]	45 [41, 49]	26 [22, 29]	1	-	
OSA, N-	26	3.5 [1.73, 12+]	88 [76, 100]	63 [46, 86]	41 [25, 69]	0.56	(0.32,0.97)	0.04
OSA, N+	42	1.78 [1.24, 12+]	67 [54, 83]	46 [32, 65]	21 [8, 52]	0.98	(0.66,1.45)	0.92
OSA, N-	26	3.5 [1.73, 12+]	88 [76, 100]	63 [46, 86]	41 [25, 69]	1	-	
OSA, N+	42	1.78 [1.24, 12+]	67 [54, 83]	46 [32, 65]	21 [8, 52]	1.75	(0.9,3.4)	0.1

OSA = Patients with obstructive sleep apnea; N- = node negative; N+ = Node positive; OS = Overall survival; HR = Hazard ratio, adjusted for age, receipt of adjuvant chemotherapy and radiation therapy. Median survival is in years. Other values are OS probabilities at 1, 2, and 5 years after surgery.

## Discussion

We find that patients with OSA are more likely than patients without OSA to have pancreatic cancers with lower TNM stage at the time of their surgical resection. The lower TNM stage was reflected in both a lower average T-stage, despite having primary tumors of equivalent size, (because local tumor invasion was less common), and a lower likelihood of having lymph node metastases. Lymph node metastases are identified in the resection specimens of up to 80% of patients who undergo surgery as first-line treatment of their primary pancreatic ductal adenocarcinoma [25, 26]. Lymph node metastases are an adverse prognostic indicator [25, 26], and are commonly present even with small (<1cm diameter) primary tumors [27]. We hypothesize that biological differences between pancreatic cancers from OSA vs. non-OSA cases explain the differences in lymph node metastases between these two groups. Although several mechanisms have been implicated in the development of lymph node metastases, [28, 29] what distinguishes pancreatic ductal adenocarcinomas that spread to lymph nodes from those that do not is poorly understood. The T-stage of a primary pancreatic ductal adenocarcinoma is defined by its size and the presence or absence of invasion into adjacent organs, and the likelihood of having local tumor invasion depends on the tumor’s proximity to adjacent organs and its biological properties. Overall, the pathological differences observed between OSA-associated and non-OSA-associated pancreatic ductal adenocarcinomas implicate OSA as influencing the progression of pancreatic cancer.

Despite the reduced likelihood of lymph node involvement and local tumor invasion noted in the OSA vs. non-OSA cases, we did not find any significant difference in overall survival between these two groups. This is not surprising for a number of reasons. First, there are many factors thought to influence patient survival after surgical resection, including tumor stage, margin status, tumor biology, the effect of chemotherapy, patient comorbidities, and overall performance status. Second, pancreatic cancer is an aggressive disease and most patients who undergo a potentially curative pancreatic resection still have a poor outcome; (median survival of patients with a resectable pancreatic cancer is less than 2 years and approximately half of all cases will develop locally advanced or metastatic disease within one year of their pancreatic resection.) As a result even prognostic factors such as lymph node involvement are not the major drivers of patient survival among patients undergoing resection of their pancreatic cancer. Third, although our results provide evidence that there are differences in the pathology between OSA and non-OSA cases, it is difficult to say that the pathology of the OSA cases is better or worse. OSA status was not associated with being more likely to have other adverse pathological factors that contribute to outcome with pancreatic cancer after surgical resection, which are margin status, tumor size, and tumor grade [25]. Finally, patients with OSA had similar trends in survival as those without OSA once nodal status was accounted for. For example, the hazard ratio for survival for OSA patients with node-positive disease compared to OSA patients with node-negative disease was 1.63. This is very similar to the hazard ratio for survival of node-positive vs. node-negative patients without OSA (HR 1.51).

The mechanism by which having OSA might influence the pathological features of a pancreatic cancer is not known. It is possible that this relationship is not directly related to having OSA but to comorbidities associated with having OSA (although we did not find any evidence for this). The potential role of OSA in influencing pancreatic cancer progression is particularly intriguing as there is considerable interest in the potential role of hypoxia in the tumor microenvironment in promoting cancer progression. A hypoxic tumor microenvironment is more likely to be acidic [30] and immunosuppressive [12], properties that are likely to affect tumor growth and progression. It is also thought that a hypoxic tumor microenvironment contributes to resistance to immune-based and conventional chemotherapy. There is considerable interest in trying to target tumor hypoxia as a therapeutic strategy by targeting some of the metabolic consequences of tumor hypoxia such as HIF-1 alpha [9]. Oxygen levels of primary pancreatic cancers obtained prior to resection indicate that the primary tumor is quite hypoxic [31], although a recent study that measured tumor hypoxia by using pre-operatively administered pimonidazole to identify areas of hypoxia in the resected pancreatic cancers found levels of hypoxia were similar to that found in other cancer types [32]. OSA results in intermittent episodes of systemic oxygen desaturation. Intermittent tissue hypoxia from apneic events in could cause further decreases in tissue oxygenation within the tumor microenvironment. The effect of such further decreases in oxygenation is not known, but could promote the selection of clones more resistant to profound hypoxia [33, 34]. In experimental models intermittent hypoxia has been found to promote preconditioning that renders cells more resistant to therapy[35]. Our study examined relationships between OSA and pathological features of pancreatic cancers; it is also possible that OSA could contribute to the development of pancreatic cancer by increasing the likelihood of having systemic disorders such as metabolic syndrome and diabetes, known risk factors for pancreatic cancer. What role tissue hypoxia resulting from OSA has on the initiation of pancreatic neoplasia is not known [36]. Overall, there is rationale for investigating if OSA induced in preclinical models affects the development and progression of pancreatic cancer as well as determining if patient OSA status adversely affects the tumor microenvironment and limits responses to cancer therapies.

Our study has certain limitations. It was a retrospective study, although this did not bias the pathology results because we used patient’s diagnostic surgical pathology records. Since OSA often goes unrecognized [7, 37], we suspect that there were a number of additional cases in our pancreatic cancer series that had undiagnosed OSA. Notably, we found a trend towards increasing prevalence of OSA diagnosis among PDAC cases in recent years perhaps because of the growing awareness of OSA over the last decade or so, and possibly because the prevalence of OSA has been increasing. Given the large size of our pancreatic cancer cohort overall relative to the likely number of such cases, we do not believe any under recognition of OSA would be sufficient to explain our findings. It would have been interesting to be able to have established the severity of OSA in all cases and how well it was being treated. The Wisconsin^2^ and Spanish Sleep [4] studies found that the relative risk of cancer was highest for patients with the most severe OSA. Our study was not powered to examine if OSA treatment could have influenced our findings. Many patients with OSA continue to be inadequately treated because of poor compliance with CPAP [19].

Overall, differences in the pathological features of pancreatic ductal adenocarcinoma observed between OSA and non-OSA cases supports the hypothesis that OSA can influence the pathological progression of pancreatic ductal adenocarcinoma.

## Supporting Information

S1 Table Characteristics of all subjects, by nodal status and OSA status. (PDF)

S2 Table Characteristics of all subjects, by whether or not patients had obstructive sleep apnea (OSA), restricted to the years 2008–2014. (PDF)
