# Supplementary material for: Obstructive Sleep Apnea and Pathological Characteristics of Resected Pancreatic Ductal Adenocarcinoma
Source: PLoS One. 2016 Oct 12;11(10):e0164195. doi: 10.1371/journal.pone.0164195 (PMC5061347; doi:10.1371/journal.pone.0164195)
Supplement: S1 Table — (PDF) [file pone.0164195.s001.pdf]

Supplemental Table 1: Characteristics of all subjects, by nodal status and OSA status

	non-OSA, N-<br>N = 210	non-OSA, N+<br>N = 752	OSA, N-<br>N = 26	OSA, N+<br>N = 43	*p* -value*
**Race - no. (%)**					
African American	20 (9.5)	41 (5.5)	1 (3.8)	4 (9.3)	
Other	24 (11.4)	65 (8.6)	1 (3.8)	3 (7)	0.23
Caucasian	166 (79)	646 (85.9)	24 (92.3)	36 (83.7)	
**Sex - no. (%)**					
Female	110 (52.4)	363 (48.3)	8 (30.8)	8 (18.6)	0.4
Male	100 (47.6)	389 (51.7)	18 (69.2)	35 (81.4)	
**Age - median (range)**	68 (35, 91)	67 (27, 92)	68 (53, 84)	67 (54, 80)	**< 0.001**
**Weight (kg) - median (range)**	73.4 (42.3, 144.4)	72.1 (36.3, 147)	79.3 (57.3, 126.3)	84.8 (55.7, 156)	0.24
**Height (m) - median (range)**	1.7 (1.4, 1.9)	1.7 (1.5, 2)	1.7 (1.4, 1.9)	1.8 (1.6, 1.9)	0.09
**BMI - median (range)**	25.4 (17.3, 36.5)	24.6 (15.1, 46.2)	26.9 (21, 45.3)	27.5 (18.7, 52.3)	0.68
**Smoking Status - no. (%)**					
Non-smoker	78 (46.4)	279 (45.4)	10 (40)	16 (38.1)	
Current	14 (8.3)	59 (9.6)	4 (16)	2 (4.8)	0.24
Former	76 (45.2)	277 (45)	11 (44)	24 (57.1)	
Unknown	42	137	1	1	
**Diabetes - no. (%)**	43 (20.5)	160 (21.3)	7 (26.9)	15 (34.9)	0.57
**Pre-Op Jaundice - no. (%)**	74 (35.2)	426 (56.6)	7 (26.9)	24 (57.1)	0.46
**Pre-Op Weight Loss - no. (%)**	72 (34.3)	294 (39.1)	8 (32)	13 (31)	0.65
**Tumor location - no. (%)**					
Body-Tail	56 (26.7)	94 (12.5)	10 (38.5)	8 (18.6)	
Head	143 (68.1)	626 (83.2)	16 (61.5)	35 (81.4)	0.94
Whole Gland	11 (5.2)	32 (4.3)	0 (0)	0 (0)	
**Tumor size - median (range)**	2.5 (0.7, 8)	3 (0.7, 9.5)	3.3 (1, 10)	3 (0.8, 8)	0.13
**Path Grade - no. (%)**					
1	16 (7.7)	20 (2.7)	1 (3.8)	2 (4.8)	
2	120 (57.4)	395 (52.7)	12 (46.2)	25 (59.5)	0.13
3	73 (34.9)	334 (44.6)	13 (50)	15 (35.7)	
Unknown	1	3	0	1	
**Vascular Invasion - no. (%)**	46 (24.7)	460 (74)	7 (31.8)	32 (78)	0.84
**Perineural Invasion - no. (%)**	172 (84.3)	684 (92.8)	24 (92.3)	39 (90.7)	0.23
**Positive Nodes - median (range)**	0 (0, 0)	3 (1, 20)	0 (0, 0)	2 (1, 16)	>0.99
**Total Nodes - median (range)**	18 (0, 51)	20 (1, 84)	18 (12, 37)	21 (3, 39)	**< 0.001**
**Nodal Ratio - median (range)**	0 (0, 0)	0.2 (0, 2)	0 (0, 0)	0.2 (0, 0.8)	>0.99
**AJCC T Stage - no. (%)**					
T1	38 (18.1)	28 (3.7)	5 (19.2)	4 (9.3)	
T2	70 (33.3)	142 (18.9)	14 (53.8)	8 (18.6)	0.24
T3	99 (47.1)	556 (73.9)	7 (26.9)	29 (67.4)	
T4	3 (1.4)	26 (3.5)	0 (0)	2 (4.7)	
**Stage - no. (%)**					
IA	38 (18.1)	0 (0)	5 (19.2)	0 (0)	
IB	70 (33.3)	0 (0)	14 (53.8)	0 (0)	
IIA	99 (47.1)	0 (0)	7 (26.9)	0 (0)	>0.99
IIB	0 (0)	726 (96.5)	0 (0)	41 (95.3)	
III	3 (1.4)	26 (3.5)	0 (0)	2 (4.7)	
**Margin Status - no. (%)**					
R0	166 (81)	492 (66)	23 (88.5)	27 (62.8)	
R1	37 (18)	232 (31.1)	3 (11.5)	16 (37.2)	0.27
R2	2 (1)	21 (2.8)	0 (0)	0 (0)	
Unknown	5	7	0	0	
**Adjuvant chemotherapy - no. (%)**	104 (49.5)	421 (56)	17 (65.4)	24 (55.8)	0.23
**Adjuvant radiation therapy - no. (%)**	58 (27.6)	278 (37)	9 (34.6)	15 (34.9)	0.45

**p* -values from logistic regression models testing for an interaction between OSA and patient characteristic on nodal status (negative vs. positive). N+ = node positive. N- = node negative
