# Supplementary material for: Obstructive Sleep Apnea and Pathological Characteristics of Resected Pancreatic Ductal Adenocarcinoma
Source: PLoS One. 2016 Oct 12;11(10):e0164195. doi: 10.1371/journal.pone.0164195 (PMC5061347; doi:10.1371/journal.pone.0164195)
Supplement: S2 Table — (PDF) [file pone.0164195.s002.pdf]

SupplementalTable 2: Characteristics of all subjects, by whether or not patients had obstructive sleep apnea (OSA), restricted to the years 2008-2014.

	non-OSA N = 509	OSA N = 55	*p* -value*
**Race - no. (%)**			
African American	32 (6.3)	4 (7.3)	
Other	59 (11.6)	4 (7.3)	0.633
Caucasian	418 (82.1)	47 (85.5)	
**Sex - no. (%)**			
Female	254 (49.9)	11 (20)	**< 0.001**
Male	255 (50.1)	44 (80)	
**Age - median (range)**	66 (27, 90)	67 (53, 84)	0.572
**Weight (kg) - median (range)**	73.3 (36.3, 147)	84.3 (55.7, 156)	**< 0.001**
**Height (m) - median (range)**	1.7 (1.4, 2)	1.8 (1.4, 1.9)	**< 0.001**
**BMI - median (range)**	25 (15.1, 45.4)	27.4 (18.7, 52.3)	**< 0.001**
**Smoking Status - no. (%)**			
Non-smoker	212 (48.8)	21 (38.2)	
Current	34 (7.8)	5 (9.1)	0.314
Former	188 (43.3)	29 (52.7)	
Unknown	75	0	
**Diabetes - no. (%)**	114 (22.4)	19 (34.5)	0.064
**Cardiovascular/cerebrovascular events - no. (%)**	45 (8.8)	9 (16.3)	0.071
**Pre-Op Jaundice - no. (%)**	222 (43.6)	21 (38.9)	0.565
**Pre-Op Weight Loss - no. (%)**	157 (30.8)	15 (28.3)	0.756
**Tumor location - no. (%)**			
Body-Tail	88 (17.3)	16 (29.1)	
Head	405 (79.6)	39 (70.9)	0.064
Whole Gland	16 (3.1)	0 (0)	
**Tumor size - median (range)**	3 (0.7, 9)	3.1 (0.8, 10)	0.355
**Path Grade - no. (%)**			
1	20 (3.9)	3 (5.6)	
2	280 (55.2)	27 (50)	0.58
3	207 (40.8)	26 (44.4)	
Unknown	2	1	
**Vascular Invasion - no. (%)**	273 (64.1)	32 (64)	>0.99
**Perineural Invasion - no. (%)**	441 (88.7)	49 (89.1)	>0.99
**Positive Nodes - median (range)**	2 (0, 20)	2 (0, 16)	**0.042**
**Total Nodes - median (range)**	21 (0, 84)	21 (9, 39)	0.454
**Nodal Ratio - median (range)**	0.1 (0, 0.8)	0.1 (0, 0.6)	**0.034**
**AJCC T Stage - no. (%)**			
T1	43 (8.4)	8 (14.5)	
T2	137 (26.9)	22 (40)	**0.016**
T3	319 (62.7)	23 (41.8)	
T4	10 (2)	2 (3.6)	
**AJCC N Stage - no. (%)**			
N0	120 (23.6)	21 (38.2)	**0.022**
N1	389 (76.4)	34 (61.8)	
**Stage - no. (%)**			
IA	23 (4.5)	4 (7.3)	
IB	48 (9.4)	14 (25.5)	
IIA	47 (9.2)	3 (5.5)	**0.005**
IIB	381 (74.9)	32 (58.2)	
III	10 (2)	2 (3.6)	
**Margin Status - no. (%)**			
R0	370 (74.1)	40 (72.7)	
R1	127 (25.5)	15 (27.3)	0.795
R2	2 (0.4)	0 (0)	
Unknown	10	0	
**Adjuvant chemotherapy - no. (%)**	259 (50.9)	31 (56.4)	0.479
**Adjuvant radiation therapy - no. (%)**	137 (26.9)	15 (27.3)	>0.99

**p* -values for Fisher's exact test for categorical variables and Wilcoxon rank sum tests for continuous variables.
